# Low dose Amantadine and Escitalopram combination in Atypical Parkinsonian disorders- A Retrospective chart review

**DOI:** 10.1192/j.eurpsy.2023.543

**Published:** 2023-07-19

**Authors:** S. M. Tripathi, P. Chutia

**Affiliations:** Department of Geriatric Mental Health, King George’s Medical University, Lucknow, India

## Abstract

**Introduction:**

The response to conventional antiparkinsonian medications is elusive in atypical parkinsonian disorders. Improvement in parkinsonian symptoms in atypical parkinsonian disorders has been reported with anecdotal use of Amantadine. The role of serotonergic control over the nigrostriatal pathway led to the use of Escitalopram.

**Objectives:**

To examine the efficacy of low dose Amantadine and Escitalopram combination treatment in atypical parkinsonian disorder

**Methods:**

A retrospective chart review of Atypical Parkinsonian disorder patients who received the combination of low dose Amantadine and Escitalopram and had follow up assessment available for a minimum of four months were studied. The primary outcome measure was change in Progressive Supranuclear Palsy rating scale, Unified Multiple System Atrophy rating scale score and Clinical Global Improvement Scale Change score in follow up visits.

**Results:**

A total of 8 patients with a mean age of 68.5 years, 6 with a diagnosis of Progressive Supranuclear Palsy(PSP) and 2 with a diagnosis of Multiple System Atrophy(MSA) met the selection criteria for the study. Patients were treated with the dose of Amantadine 50mg twice daily and Escitalopram 5mg once daily. The symptom domain to respond first was autonomic symptoms followed by gait, mentation, limb and bulbar symptoms. Amantadine has unique dopaminergic and NMDA antagonist properties. Serotonin has a role in modulation of the autonomic functions and nigrostriatal circuitry. Thus, combining Escitalopram with Amantadine can help ameliorate the array of symptoms in atypical parkinsonian disorders.

**Conclusions:**

The PSP and MSA patients responded to the combination of low dose Amantadine and Escitalopram as evidenced by objective rating scales and subjective clinician assessment. Further prospective trials for longer duration are needed to establish the effect size and stability of response.

KEYWORDS: steele richardson olszewski disease, shy dragger syndrome, antidepressants

**Disclosure of Interest:**

None Declared

